# Immediate Implants: Clinical Guidelines for Esthetic Outcomes

**DOI:** 10.3390/dj4020021

**Published:** 2016-06-13

**Authors:** Mohammad A. Javaid, Zohaib Khurshid, Muhammad S. Zafar, Shariq Najeeb

**Affiliations:** 1 Department of periodontics, University of British Columbia, Vancouver, BC V6T 1Z3, Canada; mohammad.javaid2@alumni.ubc.ca; 2 Department of Dental Biomaterials, College of Dentistry, King Faisal University, P.O. Box 400, Al-Ahsa 31982, Saudi Arabia; drzohaibkhurshid@gmail.com; 3 Department of Restorative Dentistry, College of Dentistry, Taibah University, Madina Munawarrah 41311, Saudi Arabia; MZAFAR@taibahu.edu.sa or drsohail_78@hotmail.com; 4 Department of Restorative Dental Sciences, Al Farabi Colleges, Riyadh 361724, Saudi Arabia

**Keywords:** immediate implants, clinical guidelines, esthetic dentistry

## Abstract

Research has shown that tooth loss results in morphological changes in alveolar ridge that may influence the subsequent implant placement. Immediate implant placement was introduced as a possible means to limit bone resorption and reduce the number of surgical procedures following tooth extraction. Histological and clinical evidence from human clinical studies showing efficacy of immediate implants has come to light over the last decade or so. However, immediate implant placement is a challenging surgical procedure and requires proper case selection and surgical technique. Furthermore, there appears to be a lack of clinical guidelines for immediate implant placement case selection. Therefore, the aim of this mini-review is to analyze critical evidence from human studies in order to establish clinical guidelines which may help clinicians in case selection when considering immediate implant placement protocol.

## 1. Introduction

Dental implants are surgical devices made of titanium, zirconia or polymeric materials that form a direct interface with the alveolar bone to support a prosthodontic or orthodontic appliance [1,2,3,4]. Conventionally, following tooth extraction, the alveolar socket is allowed to heal fully prior to the insertion of a dental implant [5]. However, pathological and environmental factors may prolong healing time [6]. In addition to the prolonged treatment time, it is well documented that the physiological process leads to dimensional changes in the alveolar ridge following tooth extraction such as resorption [7] during the first 3 months of healing [8]. Local factors effecting peri-implant healing include tobacco usage, periodontal disease, surgical procedure and oral hygiene, while systemic factors may include presence of systemic disease, pregnancy, *etc.* [9]. Additionally, to improve osseointegration, surfaces of dental implants are modified to enhance their bioactivity [10]. Morphological changes promoted by systemic and/or local factors in alveolar ridge can potentially influence the prognosis of implant placement [11,12].

In order to circumvent the problem of post-extraction and implant-related bone resorption, the concept of immediate implants was introduced in the late 1970s [13,14]. It was suggested that this approach could not only limit physiological bone resorption leading to better esthetic outcomes but also minimize the number of surgical procedures [15]. An initial histological study demonstrated osseointegration of immediate implants [15]. In addition, clinical evidence demonstrated the role of immediate implants in limiting physiological bone remodelling following tooth extraction [16,17]. Researchers from the University of Goteborg demonstrated that alveolar bone resorbs independent of timing of implant placement and significant dimensional changes in buccal wall take place 4–12 weeks following placement of immediate implants [7,18,19]. This was further supported by human clinical trials reporting up to 56% reduction in width of alveolar ridge following immediate implant placement [20]. In contrast to these initial results, numerous other studies published during the last 5 years report excellent survival rate, degree of osseointegration and maintenance of interdental bone levels with use of immediate implant protocol [21,22]. The immediate implant placement in infected tooth sockets may also lead to successful osseointegration [23]. The aim of the present review is to analyze critical evidence from human clinical studies on the efficacy of immediate implant placement and to provide important clinical guidelines for esthetically pleasing outcomes.

## 2. Spontaneous Healing *vs.* Immediate Implant Healing

Very limited research has been published comparing healing of extraction sockets with and without dental implants. One study reported similar reduction in bone height in implant and edentulous sites after 3 months of healing [24]. Vignoletti and coworkers [25] reported significantly greater vertical bone loss in immediate implant sites compared to edentulous sites. These studies clearly demonstrate that immediate implant placement protocol does not prevent bone resorption following tooth extraction.

## 3. Hard Tissue Changes after Immediate Implant Placement

Botticelli and colleagues [20] reported that the buccal bone plate undergoes more than 50% reduction in horizontal dimensions following placement of single unit immediate implant in maxilla. Similar findings were observed in another clinical trial [26] where cylindrical and conical shaped implants were placed in extraction sockets. Both groups showed 36% reduction in width in buccal bone wall. The authors also reported a mean vertical bone loss of 1 mm that was accentuated in the presence of a thin buccal wall and placement of implants in anterior maxilla. Multivariate analyses revealed that the thickness of buccal bone wall was a key factor influencing horizontal bone resorption changes. Similarly, vertical changes were significantly influenced by implant position and the thickness of buccal bone wall [26].

Various approaches have been proposed to counter these hard tissues dimensional changes. Most of these strategies involve combination of immediate implant placement with simultaneous use of various grafting materials and barrier membranes [27]. Chen *et al.* [27] analyzed placement of immediate implants in maxilla. Bone grafts with or without membrane were used to fill the gap between implant and inner bone surface in test groups. No bone graft material was used in the control group and the gap between implant and inner bone surface was left unfilled. The experimental groups showed significantly reduced horizontal resorption compared to the control group. Vertical resorption, however, was similar among the groups and was influenced by the thickness of the buccal bone plate [27]. Recently, clinicians have observed more stable placement of immediate non-functional restorations when combined with bone grafting [28,29]. A recent study by Romao *et al.*, suggests that laser-induced biostimulation may improve bone repair post-extraction [30]. However, the effect of biostimulation has not been investigated in sites of immediate implant placement.

## 4. Soft Tissue Changes Following Immediate Implants

Recently, a systematic review analyzing recession associated with immediate implants has been published [31]. Marginal tissue recession of at least 1 mm was reported in studies with observation period of 3 years or more. Such untoward clinical outcome was observed in 20% of the patients, but this observation was made in just two studies with no control groups. Factors that influenced marginal tissue recession include [31]:

(a) Position of implant, with greater recession being a common occurrence when the implants were positioned buccally.
(b) Gingival biotype—increased recession was observed in cases with thin biotype.

Similar results were reported by Chen *et al.* [27] demonstrating at least 1 mm recession in over 30% of the sites after 18 months of follow up period. The authors also reported significant association between marginal recession and position of implant in relation to buccal bone plate. Recession was seen in 16.7% of the implants placed lingually as compared to 58.3% of the buccally placed immediate implants. Bianchi and Sanfilippo [32] assessed the added value of connective tissue grafts in conjunction with immediate implant. They compared the mucosal marginal level following installation of final restoration and compared the marginal level with adjacent teeth. They observed that all patients who received connective tissue grafts with immediate implants showed less than 1 mm of marginal tissue discrepancy. This outcome was achieved only in 80% of the subjects who received immediate implants alone. Canullo *et al.* [33] studied the use of platform switch implants in context of marginal tissue recession. They reported significantly less recession when platform switch implants were used. However, a recent systematic review by Lee *et al.*, has not found any significant advantage of using connective tissue grafts towards reducing gingival recession [34]. Hence, more studies are required to advocate the combined use of soft tissue grafts and immediate implants. Provisional restorations, following immediate placement of implants, may also improve soft-tissue healing [35]. It has been observed that placement of provisional crowns on immediately placed implants may not only improve preservation of buccal bone, but also improve esthetics by reducing gingival recession [36,37].

## 5. Clinical Guidelines and Conclusions

The clinical guidelines for immediate implant placement protocol are summarised in Table 1.

Based on the evidence, thickness and integrity of buccal bone plate and gingival biotype are the critical factors that play a pivotal role in success of immediate implants. When following immediate implant protocol, buccal position of the implants should be avoided. Implants should be slightly palatally/lingually placed. The gap between implant and inner bone surface should be filled with bone substitutes which have a low resorption rate [38]. In order to compensate for the expected vertical resorption, implant should be placed at least 1 mm apical to buccal ridge or 2–3 mm from gingival margin [27]. In addition, factors such as use of platform switch implants, flapless surgical approach, simultaneous placement of connective tissue grafts and immediate provisional restorations may also be considered. Success with immediate implant protocol requires advanced surgical skills, ideal extraction socket conditions and knowledge of local anatomy. It is recommended that when ideal conditions are not present, other implant timing protocols that have provided excellent clinical outcomes with regards to soft and hard tissues should be followed [39].

## Figures and Tables

**Table 1:** Clinical Guidelines for Esthetic Outcomes When Using Immediate Implant Protocol.

Thick and Intact Buccal Bone Wall
Thick gingival biotype
Minimal trauma in tooth extraction
Presence of at least 3 socket walls—ideally 4 walls
Implant shoulder should be placed 2–3 mm apical to anticipated gingival margin
Primary implant stability with engagement of 3–4 mm bone apical to root apex
Slight palatal/lingual positioning of implant
Fill the gap between implant and inner bone surface using a low resorbing bone graft material with or without membrane
